# Tooth shape and sex estimation: a 3D geometric morphometric landmark-based comparative analysis of artificial neural networks, support vector machines, and Random Forest models

**DOI:** 10.1007/s13205-025-04439-7

**Published:** 2025-07-26

**Authors:** Srikant Natarajan, Junaid Ahmed, Ruban Sundarraj, Varenya Vinay, Shravan Shetty, Nidhin Philip Jose, Sharada Chowdappa, Sunitha Carnelio

**Affiliations:** 1 Department of Oral Pathology and Microbiology, Manipal College of Dental Sciences, Mangalore, India; 2 Manipal Academy of Higher Education, Manipal, Karnataka India 576104, India,; 3 Department of Software Technology, St Aloysius (DEEMED TO BE UNIVERSITY) Institute of Management and IT- (AIMIT), Beeri, Mangaluru, Kotekar 575022 India; 4 Department of Orthodontics and Dentofacial Orthopaedics, Manipal College of Dental Sciences, Mangalore, Karnataka India; 5 Private Dental Researcher, Mangaluru, India; 6 Department of Oral Pathology and Microbiology, Manipal College of Dental Sciences, Manipal, India; 7 Department of Oral Medicine and Radiology , Manipal College of Dental Sciences, Mangalore , India

**Keywords:** Forensic Anthropology, Sex estimation, Machine learning, 3D geometric morphometrics, Dental landmarks, Random Forest algorithm, Forensic odontology

## Abstract

This study evaluated the performance of three artificial intelligence (AI) algorithms—support vector machine (SVM), artificial neural network (ANN), and Random Forest (RF)—in sex estimation using 3D geometric morphometric data derived from nine permanent tooth classes in 120 individuals (60 males, 60 females). Dental casts from 60 males and 60 females, aged 13–20 were digitized using a 3D scanner. Anatomic and geometric landmarks were identified on nine tooth types (maxillary/mandibular premolars and molars) via 3D Slicer software. Landmark coordinates underwent Procrustes superimposition and principal component analysis. Three AI models (ANN, SVM, RF) were trained on pre-processed landmark data, with performance evaluated using fivefold cross validation, accuracy, precision, recall, F1-score, and AUC. RF outperformed SVM and ANN across all tooth types, achieving the highest accuracy (97.95% for mandibular second premolars) and balanced precision/recall (0.85–1.0). SVM showed moderate performance (70–88% accuracy), while ANN had the lowest metrics (58–70% accuracy). Maxillary first molars (95.83% accuracy) and mandibular second premolars (97.95%) exhibited the highest sexual dimorphism. RF demonstrated minimal sex bias, whereas ANN struggled with female classification (recall: 0.33–0.88 vs. males: 0.36–1.0). Feature analysis highlighted mandibular premolars as most dimorphic, with RF leveraging complex spatial relationships between landmarks effectively. Random Forest emerged as the most robust model for sex estimation using 3D dental landmarks, likely due to its ability to handle tabular data and high-dimensional feature spaces. Traditional machine learning models (RF, SVM) outperformed ANN, suggesting data set structure and feature engineering influence AI efficacy. These findings underscore AI’s potential to enhance objectivity and accuracy in forensic odontology, particularly with geometric morphometric data. Future research should explore hybrid models combining AI strengths with traditional morphometrics for improved reliability.

## Introduction

Sex estimation is a critical step in establishing the biological profile of unidentified human remains in forensic investigations. Traditional methods often rely on morphological characteristics of the skull, face and teeth for identification. Odontometric methods, which involve the measurement of teeth, often play an essential role in estimating sex, particularly in cases involving young individuals or fragmented remains. Teeth are highly resistant to various post-mortem insults, making them a valuable tool in forensic anthropology. While traditional odontometric approaches often involve linear measurements, the advent of three-dimensional imaging techniques, such as cone beam computed tomography (CBCT), photogrammetry and three-dimensional (3D) surface scanners, allows for the acquisition of more comprehensive quantitative data on tooth dimensions as well as shape, overcoming limitations associated with conventional radiographs. (Zhang and Maga 2017; Wang et al. 2019; Ajmal et al. 2023).

The shape of a tooth has traditionally been described subjectively using terms, such as rectangular, ovoid, or hexagonal, based on various viewing perspectives. However, these descriptions inherently involve a subjective component and exhibit poor interobserver reliability. (Horvath et al. 2012) To overcome this limitation, landmark-based analysis provides a more precise and objective method for representing, visualizing, and quantifying tooth shape. The advent of geometric morphometry, combined with powerful statistical tools such as multivariate analysis of variance and dimensional reduction techniques like principal component analysis, has significantly improved the accuracy and depth of shape evaluation, offering a more robust framework for dental morphology studies. (Al-Shahrani et al. 2014; Choi et al. 2020; Natarajan et al. 2022) With the advent of open source communities these techniques and softwares involved are evolving very fast. Softwares such as Slicer 3D, Morpho J, Thin Plate Spline Software Suite (TPS) and PAleontological STatistics Software Package (PAST) are freely available for performing geometric morphometry analysis. (Klingenberg 2011; Rolfe et al. 2021) These techniques require considerable human effort in acquisition of two-dimensional (2D) or 3D images and accurately marking landmarks. Furthermore, the process involves a learning curve for researchers to grasp the complexities of softwares involved and the advanced statistical analysis and interpretation procedures.

In recent years, there has been a growing interest in applying artificial intelligence (AI) and machine learning techniques to enhance the accuracy and efficiency of sex estimation in forensic odontology. (Franco et al. 2022) AI algorithms, such as artificial neural networks (ANNs) and support vector machines (SVM), have shown promise in recognizing complex patterns in biological data for sex classification. (Esmaeilyfard et al. 2021; Bianchi et al. 2023) These methods can analyze various features, including dental measurements obtained from radiographs or three-dimensional scans, to predict an individual's sex. Previous research has explored the potential of machine learning for sex estimation using odontometric parameters derived from CBCT images, achieving high classification accuracies. (Esmaeilyfard et al. 2021; Khanagar et al. 2021) Furthermore, studies have also investigated the use of AI with geometric morphometric analysis of tooth shape for sex determination, often demonstrating improved results compared to traditional odontometric methods alone. The first molar teeth have been specifically investigated for their potential in sex prediction using traditional methods and, more recently, data mining approaches. (Franco et al. 2022; Bianchi et al. 2023).

Based upon the established significance of odontometric analysis and the increasing application of AI in forensic sex estimation, this study aims to develop and evaluate the performance of three different artificial intelligence algorithms—support vector machine (SVM), artificial neural network (ANN), and Random Forest—for sex differentiation using three-dimensional landmark data collected from nine teeth classes. An additional objective is to assess whether the application of AI enhances the efficiency and accuracy of sex estimation compared to conventional statistical approaches.

## Materials and methods

### Sample size calculation

A previous study by Yong et al. (2018) demonstrated a significant difference in the shape of human premolars between males and females. (Yong et al. 2018) The centroid sizes of the upper premolars were found to be significantly larger in males (first premolar: 28.33 ± 1.96; second premolar: 27.54 ± 1.83) than in females (first premolar: 27.82 ± 1.93; second premolar: 26.93 ± 2.05). Based on these values, the sample size was determined using equation for comparing means between the two groups:

$\mathbf{N}=\frac{2{{({{\varvec{Z}}}_{1-\frac{\boldsymbol{\alpha }}{2}}+{{\varvec{Z}}}_{1-{\varvec{\beta}}})}^{2}{\varvec{\sigma}}}^{2}}{{{\varvec{d}}}^{2}}$, where N is the sample size, d is the clinically significant difference, and σ is the mean standard deviation. Considering the centroid values, a 5% alpha error (z = 1.96), 80% power (z = 0.84), and a clinically significant difference of 1 unit, the required sample size per sex was calculated to be 60 individuals.

### Sample collection and inclusion criteria

Prospective pretreatment dental casts were obtained from the archives of the Department of Orthodontics at the Manipal College of Dental Sciences, Mangalore, in accordance with the sample size calculation. Our requirement was 120 casts accounting for 60 males and 60 females. However, to ensure anatomical variation, an additional 40 casts were examined, making the total sample size of 160 casts, from which 60 male and 60 female casts were selected for analysis. Clearance was obtained from the institutional ethics committee prior to the initiation of the study.

Study casts of individuals aged 13–20 years were included to prevent tooth changes arising from occlusal wear and attrition, which could lead to inaccurate landmark acquisition. Comprehensive demographic histories were recorded, including birthplace and domicile information for three generations, ensuring regional consistency of origin of the participants. Patients having full complement of posterior teeth from first premolar to second molar in maxilla and mandible bilaterally were selected. Individuals having developmental anomalies, syndromes or with posterior teeth having restorations, fractures, caries or crowns were excluded from the study.

### Digital acquisition and landmark identification

Impressions were taken using Dentsply Aquasil Soft Putty and poured with Type 4 Extra Hard Dental Die Stone (Zhermack, Italy). The casts were digitized using the inEOS X5-Lab scanner (Dentsply Sirona, Noida, India). Landmarks were identified using anatomic and geometric evidence, as described by Biggerstaff (1969), Robinson et al. (2002), and Al-Shahrani et al. (2014). (Biggerstaff 1969; Robinson et al. 2002; Al-Shahrani et al. 2014) Landmarks were classified into anatomic (based on cusp tips, fissure junctions, mesiodistal and buccolingual width endpoints, line and point angles) and geometric (based on crests of curvatures, occlusal surface landmarks) evidences. 3D landmarks were marked using 3D Slicer software (version 4.10.2, http://www.slicer.org, accessed on 25th December 2023)(Rolfe et al. 2021).The number of landmarks varied based on tooth complexity, with mandibular first premolars, second premolars (two- and three-cusp types), first molars, and second molars containing 20, 19, 21, 32, and 27 landmarks, respectively. The maxillary first premolar, second premolar, and molars had 20, 19 and 28 landmarks, respectively, based on anatomic and geometric evidence. The figures and detailed descriptions of these landmarks have been provided in our previous research. (Natarajan et al. 2024a, b).

Each of the landmarks were recorded in 3D- Slicer software and tabulated in Microsoft Excel in x, y, z format, representing the coordinates in the three axis. These data were imported into MorphoJ version 1.07a software to perform procrustes superimposition after averaging the left and right coordinates by individual. Conventional statistics was performed using the averaged coordinate data to evaluate variation in shape between sexes using principal component analysis, procrustes ANOVA and discriminant function analysis.

The landmark coordinates were next imported into AI-based classification models. Three machine learning algorithms were employed: artificial neural networks (ANN), support vector machines (SVM), and Random Forest (RF). The classification process was conducted in the AI research lab at St Aloysius Deemed to be University and involved the following steps:

**Preprocessing and normalization**: For preprocessing, data were extracted by selecting the Gender and Left/Right columns. Pairwise distances were computed to transform raw data coordinates to distance variables as a part of normalization. The data set was then reshaped to represent 3D landmark coordinates. Since this is a geometric morphometrics data set, we measured pairwise distances between landmarks to capture the spatial relationships between anatomical points. By calculating all possible distances between these landmarks, we quantified the geometric structure of the tooth based on the principles of procrustes superimposition. In addition, Mahalanobis distance was computed to evaluate the differences between shapes in shape space. For feature selection, a *t* test was performed to identify features that significantly differed between genders, and the data set was further augmented to enhance model training.

### Model building and evaluation

For model development, the data set was divided into 80% training and 20% testing. Three models were tested: Random Forest, support vector machine (SVM), and an artificial neural network (ANN). To evaluate model performance and detect overfitting, a fivefold cross-validation test was conducted, ensuring robustness and reliability in classification accuracy. Given the aim of this research was to identify sex based on geometric morphometric features, three AI algorithms were chosen, i.e., tree-based classifier–Random Forest, margin-based classifier–support vector machine (SVM) and deep learning-based classifier–artificial neural network.

Random Forest is known for its ability to handle high-dimensional feature spaces, it is robust to noise and has the capability to capture complex interactions between distance-based features. SVM with a linear kernel was included as it is effective in higher dimensional classification problems due to its strong decision boundaries for distinguishing between male and female tooth structures. For the ANN, a feedforward multilayer perceptron architecture was used. Standard settings optimized for tabular classification tasks with 500 epochs, learning rate of 0.001, with a stochastic gradient-based optimizer were applied to ensure baseline performance. Artificial neural network has the ability to learn intricate patterns and non-linear relationships making it a strong AI model for gender classification by tooth landmarks.

To test the performance of these models, Accuracy, AUC (Area Under the Curve), Precision, Recall, F1-score was chosen. In addition, a fivefold stratified cross validation was performed to obtain reliable performance metrics.

To assess the generalizability of the best-performing model, an external validation was conducted using an independent data set. The best performing models trained on the internal data set for the maxillary first and second molars were applied to their respective unseen test sets. Each model was evaluated on a separate set of 86 independent samples, providing a robust measure of performance beyond internal cross validation.

## Results

Following procrustes superimposition to normalize the landmarks and principal component analysis to reduce the dimensions, discriminant function analysis based on 3D geometric morphometric landmarks was used to classify sex, revealing varying prediction accuracies across different teeth. In the maxillary segment, the highest accuracy was observed in the first molar, with an overall prediction accuracy of 95.83% (96.67% for females and 95% for males) and a Mahalanobis distance of 3.294 (*p* = 0.0751). Similarly, the second molar showed high accuracy at 95.00% (93.33% for females and 96.67% for males) with a Mahalanobis distance of 3.0694 (*p* = 0.1735). Both the first and second molars did not show statistical significant differences; however, the premolar segment showed significant discriminating power. The lowest accuracy was recorded for the second premolar, which had an overall accuracy of 87.50% (91.67% for females and 83.33% for males) with a Mahalanobis distance of 2.24 (*p* = 0.0148). The Maxillary first premolar followed closely with an accuracy of 89.17% (86.67% for females and 91.67% for males) and a Mahalanobis distance of 2.4315 (*p* = 0.0080) (Table 1).

**Table 1:** Accuracy of 3D geometric morphometric analysis using discriminant function analysis

	Variance %	Procrustes distance	Mahalanobis distance	T-square	P value	Prediction accuracy Female	Prediction Accuracy Male	Overall Accuracy
Maxillary First Premolar	0.01055685	0.02289014	2.4315	177.3690	**0.0080**	86.67%	91.67%	89.17%
Maxillary Second Premolar	0.00799373	0.0196	2.24	150.5316	**0.0148**	91.67%	83.33%	87.50%
Maxillary First Molar	0.01351701	0.022925	3.294	325.5115	0.0751	96.67%	95%	95.83%
Maxillary Second Molar	0.02557597	0.028369	3.0694	282.6381	0.1735	93.33%	96.67%	95.00%
Mandibular First Premolar	0.01940016	0.02361	1.8882	106.96	0.3181	83.333%	78.333%	80.833%
Mandibular Second Premolar (Two cusp type)	0.02558056	0.0472	6.8036	547.91	0.94	95.00%	100%	97.95%
Mandibular Second Premolar (Three cusp type)	0.01443143	0.0224	2.7051	198.3	**0.019**	90.625%	91.49%	90.99%
Mandibular First Molar	0.00749010	0.0205	3.7158	414.21	0.3073	95.00%	95.00%	95.00%
Mandibular Second Molar	0.00976514	0.0251	3.0236	274.27	0.1063	95.00%	88.33%	91.66%

Note: Values less than p = 0.05 are considered statistically significant and are shown in bold

In the mandibular segment, the highest accuracy was achieved by the two-cusp second premolar, with a perfect prediction accuracy of 100% for males and 95.00% for females, leading to an overall accuracy of 97.95% correlating with a higher Mahalanobis distance of 6.8036 (*p* = 0.94). The first molar also exhibited high accuracy at 95.00% for both sexes, with a Mahalanobis distance of 3.7158 (*p* = 0.3073). The lowest accuracy was recorded for the first premolar, which had an overall accuracy of 80.83% (83.33% for females and 78.33% for males) with a Mahalanobis distance of 1.8882 (*p* = 0.3181). The second molar showed an overall accuracy of 91.66% (95.00% for females and 88.33% for males) with a Mahalanobis distance of 3.0236 (*p* = 0.1063). Comparatively, premolars demonstrated higher sexual dimorphism than molars, and mandibular second premolars showed the highest accuracy across all teeth (Table 1).

**Fig. 1:**
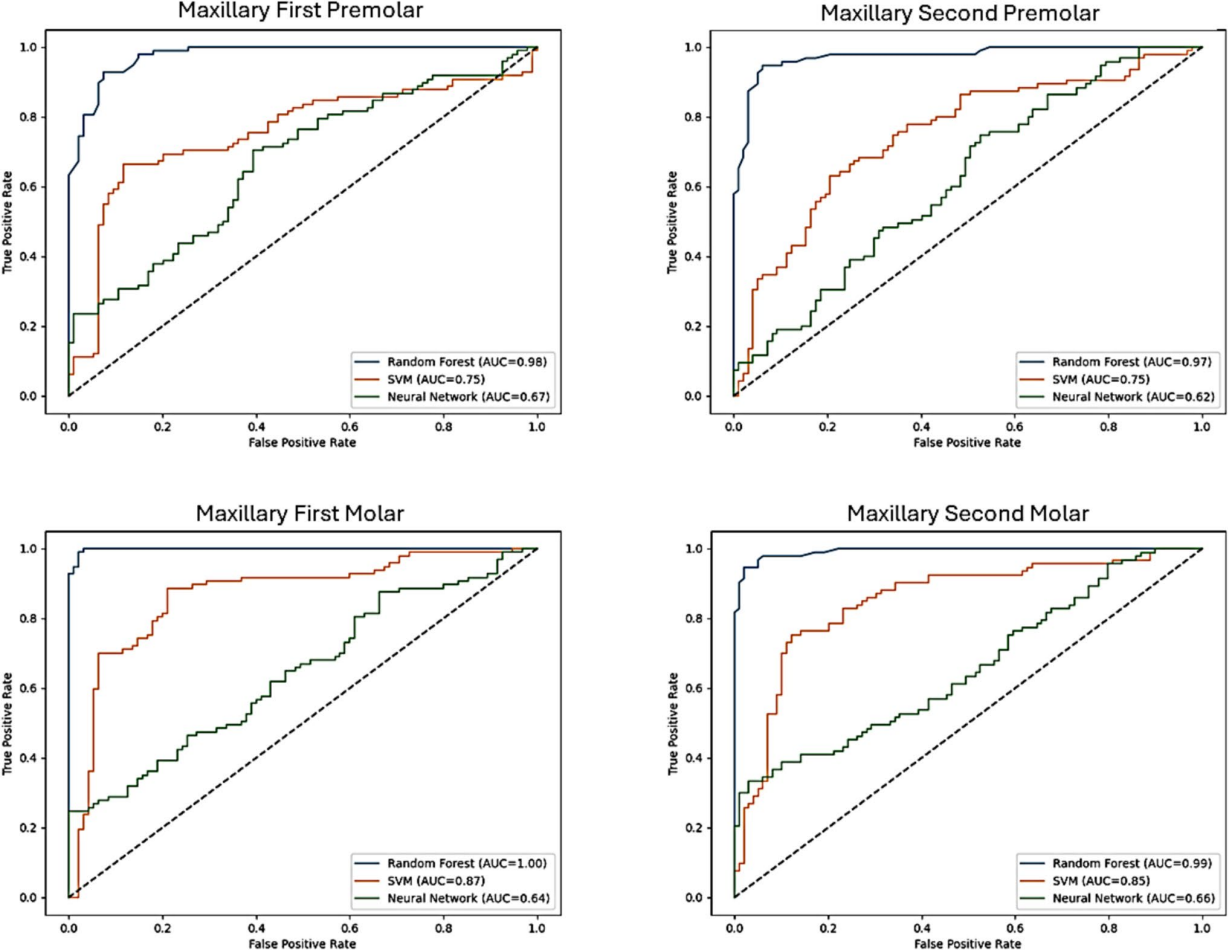
Receiver operating characteristic (ROC) curves depicting the classification performance of three machine learning models—artificial neural network (ANN), support vector machine (SVM), and Random Forest (RF)—for sex estimation based on geometric morphometric landmark data from maxillary premolars and molars. The area under the curve (AUC) values are shown for each model, reflecting their accuracy in differentiating between male and female subjects for each maxillary tooth type. Random Forest shows the highest discriminatory power, indicating superior classification performance the teeth

**Fig. 2:**
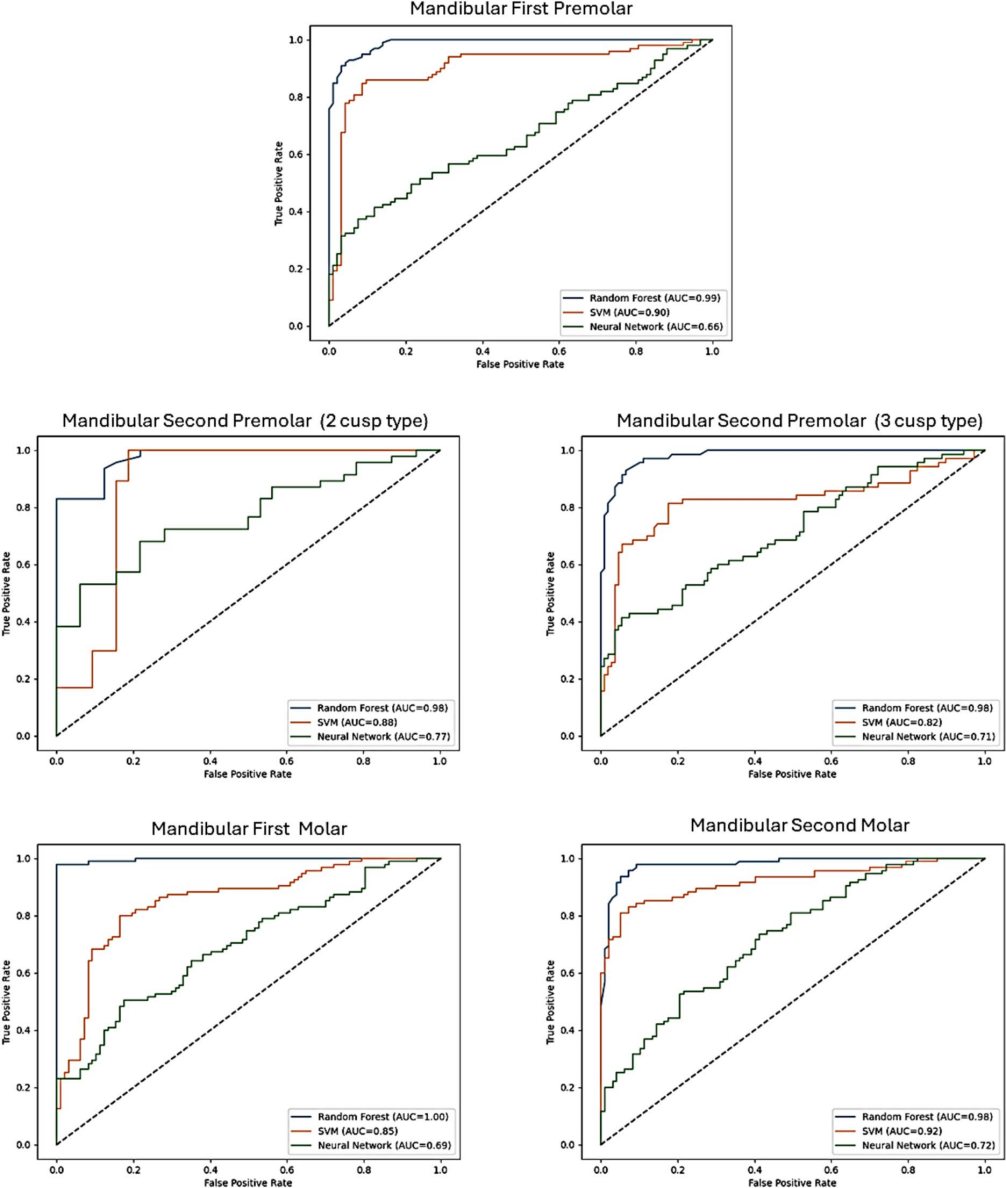
Receiver operating characteristic (ROC) curves illustrating the classification performance of ANN, SVM, and RF models for sex prediction using landmark data from mandibular premolars and molars. AUC values are provided for each model and tooth type, indicating their respective discriminative abilities. Random Forest shows the highest discriminatory power, indicating superior classification performance the teeth

The performance of the three classification models—artificial neural network (ANN), support vector machine (SVM), and Random Forest (RFM)—varied across different tooth types in terms of precision, recall, and accuracy. For ANN, precision ranged from 0.51 to 0.81, recall varied from 0.33 to 0.88, and accuracy spanned 58% to 70% across all teeth. SVM exhibited a slightly better performance, with precision values between 0.67 and 0.87, recall ranging from 0.6 to 0.93, and accuracy falling between 70 and 88%. Random Forest outperformed both models, with precision ranging from 0.85 to 1.0, recall between 0.88 and 1.0, and accuracy spanning 89% to 99%, making it the most robust classifier among the three. In the analysis of sex classification using tooth landmarks, the artificial neural network (ANN) method showed the highest precision for maxillary teeth in the first premolar (0.67 for females) and for mandibular teeth in the first premolar (0.79 for males). The highest recall using ANN was observed in the maxillary first premolar for males (0.78) and in the mandibular first premolar for females (0.88). For the support vector machine (SVM) model, the highest precision in maxillary teeth was for the maxillary second premolar (0.74 for males), while in mandibular teeth, the highest precision was found in the second premolar (three-cusp type) (0.84 for females). The highest recall using SVM was in the maxillary second premolar for females (0.79) and in the mandibular second premolar (three-cusp type) for males (0.85). The Random Forest (RFM) model consistently demonstrated high precision, with the maxillary first molar (0.98 for males) and mandibular first molar (1.0 for males) achieving the highest values. The highest recall in the maxillary arch using RFM was in the first molar for males (0.98), while in the mandibular arch, the first molar for males showed the highest recall (1.0) (Table 2, Figs. 1, 2).

**Table 2:** Performance of Artificial Neural Network (ANN), Random Forest (RF), and Support Vector Machine (SVM) models in predicting sex classification using tooth landmarks

	Model	Accuracy	Precision		Recall		F1-score	
			Female	Male	Female	Male	Female	Male
Maxillary First Premolar	ANN	0.62	0.67	0.6	0.47	0.78	0.55	0.68
	RF	0.92	0.9	0.94	0.94	0.9	0.92	0.92
	SVM	0.7	0.69	0.71	0.7	0.7	0.7	0.71
Maxillary Second Premolar	ANN	0.58	0.68	0.55	0.33	0.84	0.44	0.67
	RF	0.94	0.94	0.94	0.94	0.94	0.94	0.94
	SVM	0.7	0.67	0.74	0.79	0.6	0.73	0.66
Maxillary First Molar	ANN	0.58	0.65	0.56	0.34	0.83	0.44	0.67
	RF	0.97	0.96	0.98	0.98	0.96	0.97	0.97
	SVM	0.81	0.82	0.8	0.79	0.82	0.8	0.81
Maxillary Second Molar	ANN	0.58	0.59	0.56	0.59	0.57	0.59	0.57
	RF	0.96	0.95	0.97	0.97	0.95	0.96	0.96
	SVM	0.79	0.81	0.77	0.77	0.81	0.79	0.79
Mandibular First Premolar	ANN	0.64	0.59	0.79	0.88	0.41	0.7	0.54
	RF	0.94	0.93	0.95	0.95	0.93	0.94	0.94
	SVM	0.87	0.83	0.91	0.91	0.83	0.87	0.87
Mandibular Second Premolar (Two cusp type)	ANN	0.7	0.6	0.81	0.78	0.64	0.68	0.71
	RF	0.89	0.85	0.91	0.88	0.89	0.86	0.9
	SVM	0.87	0.87	0.88	0.81	0.91	0.84	0.9
Mandibular Second Premolar (Three Cusp type)	ANN	0.61	0.71	0.51	0.6	0.63	0.65	0.56
	RF	0.93	0.94	0.91	0.94	0.91	0.94	0.91
	SVM	0.81	0.84	0.76	0.85	0.74	0.85	0.75
Mandibular First Molar	ANN	0.62	0.58	0.74	0.88	0.36	0.7	0.48
	RF	0.99	0.98	1	1	0.98	0.99	0.99
	SVM	0.79	0.76	0.81	0.84	0.74	0.8	0.77
Mandibular Second Molar	ANN	0.66	0.69	0.63	0.59	0.73	0.63	0.68
	RF	0.94	0.97	0.91	0.91	0.97	0.94	0.94
	SVM	0.88	0.85	0.92	0.93	0.83	0.89	0.87

Comparing the three models based on F1-score, Random Forest outperformed both ANN and SVM in almost all tooth groups, demonstrating superior performance in both maxillary and mandibular teeth. The highest F1-score for ANN was seen in the maxillary first premolar (0.68 for males) and mandibular second premolar (two-cusp type) (0.71 for males). For SVM, the highest F1-score in the maxillary teeth was observed in the maxillary first premolar (0.71 for males), while in the mandibular arch, the second premolar (two-cusp type) showed the highest F1-score (0.90 for males). Random Forest achieved the highest F1-scores overall, with the mandibular first molar (0.99 for both sexes) and maxillary first molar (0.97 for males) demonstrating the best results, confirming its superiority over ANN and SVM (Table 2, Figs. 1, 2).

**Fig. 3:**
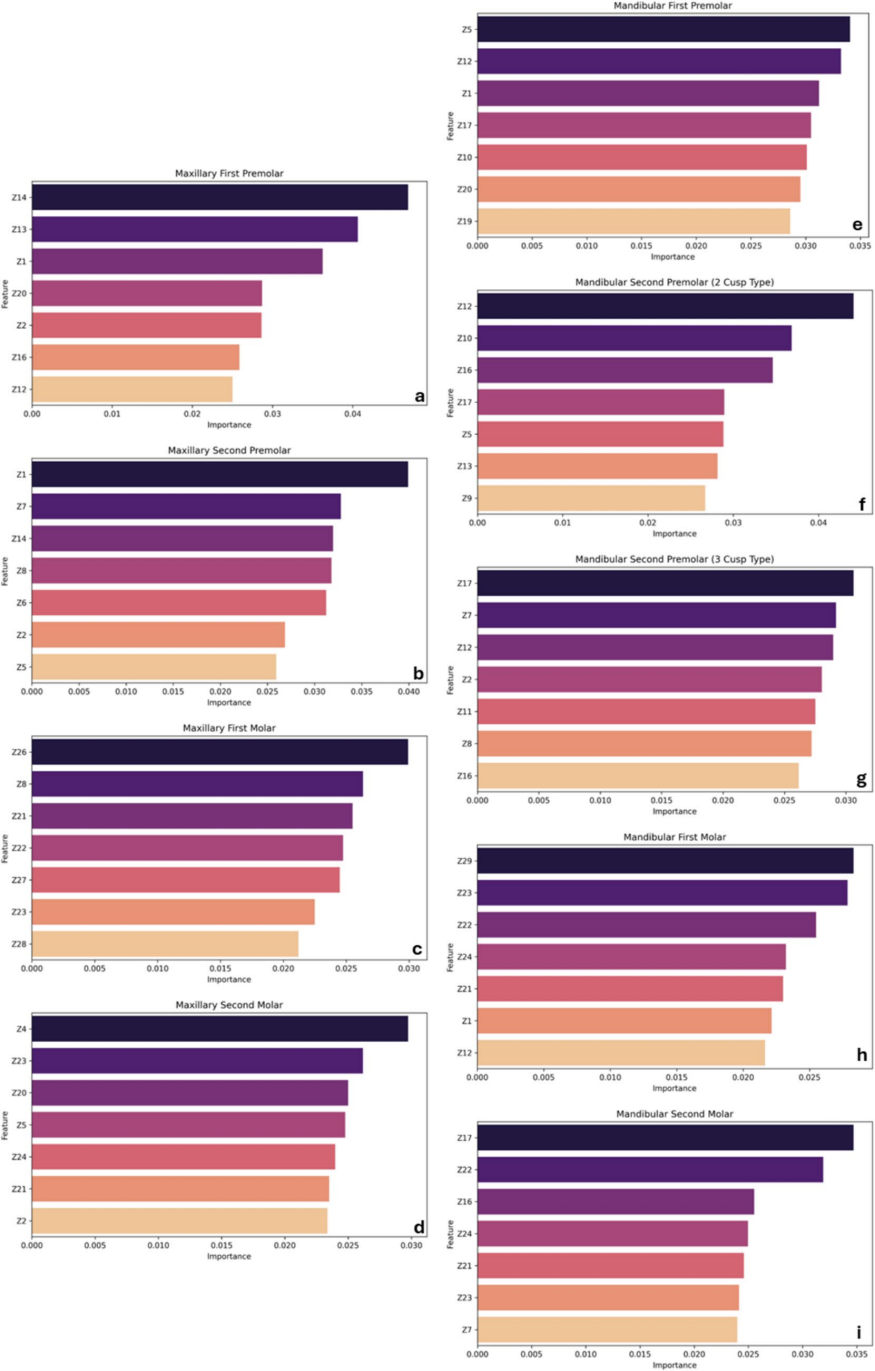
Panels **a**–**i** depict the feature importance plots generated using Random Forest (RF) analysis for individual maxillary and mandibular teeth

To enhance model interpretability, feature importance was computed using the Random Forest classifier for each tooth. This analysis identified which landmarks had the greatest influence on sex classification. The plots revealed that *z*-coordinates contributed more than x and y coordinates. In each category, the top 7 most important features were from the *z*-axis, indicating that the depth or the vertical position of anatomical landmarks had the highest discriminative power. This highlights the value of using 3D analysis in geometric morphometry. (Fig. 3a–i).

## Discussion

In this study, we evaluated the effectiveness of different artificial intelligence (AI) algorithms in sex estimation and found that Random Forest (RF) and support vector machine (SVM) outperformed artificial neural networks (ANN) in distinguishing sex. These findings contrast with some previous studies that have reported the high accuracy of ANN and Convolutional Neural Networks (CNN) in similar forensic applications like the landmark-based analysis studied by Bianchi I et al. (Bianchi et al. 2023) and Oliva G et al. (Oliva et al. 2021) in Italian population. Our findings align with the study by Esmaeilyfard et al. (2021), which reported strong performance for RF (accuracy of 85.67%) and SVM (86.97%) in sex classification based on linear tooth dimension measurements from CBCT images. Esmaeilyfard et al.’s study also found that Naïve Bayesian (NB), a probabilistic classifier performed even better (accuracy of 89.35%), than the RF and SVM algorithms, likely due to resistance to overfitting. (Esmaeilyfard et al. 2021) Naïve Bayesian classifiers are particularly useful for tooth landmarks for its simplicity and treating each tooth position as independent feature and can thus be explored in future studies. Conversely, studies such as those by Vila-Blanco et al. (2020) and Porto et al. (2020) emphasize the effectiveness of CNNs and ANNs, especially in analyzing complex morphological structures in estimating chronological age and sex using radiographs and facial photographs. (Porto et al. 2020; Vila-Blanco et al. 2020).

One possible explanation for the superior performance of SVM over ANN in this study is that the data set characteristics, including feature selection and sample size, might have favored these traditional machine learning models. Support vector machines are algorithms that generate a set of hyperplanes to classify the data into categories depending on the type of classification problem. (Nikita and Nikitas 2020) A hyperplane is a plane defined in the multidimensional space. In case of this study, the multiple landmarks with the use of principal component analysis leads to a shape space in multiple dimensions. As SVM is tuned to optimally perform in multiple dimensions, it proved superior for landmark data to classify sex. Nikita and Nikitas (2020) have studied the classification system of various algorithms in craniometric data and they found that SVM performs as well as the linear discriminant analysis in their data set but they found that RF was inferior in classification. On the contrary, in this study, the RF algorithm performed better in classifying sex. Random forest works by creating multiple decision trees using random samples of the training data. At each split in a tree, a random subset of predictors is considered instead of using all predictors. The trees are grown fully without pruning. Finally, the predictions from all trees are combined, usually by averaging (for regression) or majority voting (for classification), to improve accuracy and reduce overfitting. (Nikita and Nikitas 2020).

An artificial neural network (ANN) is a system of connected neurons that mimics how the human brain processes information to find patterns in data. It has multiple layers: the input layer, which takes in data, hidden layers that process the information, and the output layer, which provides results. The hidden layers are called"hidden,"because their values are not directly present in the training data. The network learns by adjusting weights assigned to inputs using different training algorithms to improve accuracy. (Nikita and Nikitas 2020).

Regardless of the specific AI model used, AI-driven approaches offer several advantages over traditional sex estimation methods. AI models have demonstrated superior accuracy and precision, often matching or exceeding the performance of trained human examiners. Traditional morphometric and odontometric techniques rely heavily on human expertise, leading to variability and subjectivity in assessments. AI mitigates this issue by providing objective and reproducible analyses. Esmaeilyfard et al. (2021) evaluated linear measurements in CBCT images for sex estimation and found Genetic algorithm combined with Naïve Bayesian algorithm achieved an average classification accuracy of 92.31% for sex prediction. (Esmaeilyfard et al. 2021). Anic-Milosevic et al. (2023) evaluated the dimensions of canine and the odontometric ratios to classify sex and found an accuracy of > 80% by utilizing ANN models. (Anic-Milosevic et al. 2023).

A CNN-based approach, another machine learning algorithm for sexual dimorphism showed positive outcomes with high classification accuracy in facial analysis. Verma et al. (2021) showed that the use of logistic regression and CNN showed a high AUC of 0.94 in sex estimation using 2000 images of males and females. (Verma et al. 2021) In this study, ANN generally performed better for males, especially in recall, where it struggled to correctly identify females. In contrast, SVM showed relatively balanced performance between sexes but demonstrated a bias toward males in precision scores. Random Forest showed consistent performance across sexes with minimal variation. In the mandibular first molar, both males and females achieved an F1-score of 0.99.

Since the best performing model was the Random Forest classifier, we evaluated the model using extra samples as a part of external validation. For the maxillary first molar, the Random Forest classifier achieved an overall accuracy of 85%, with precision and recall values of 0.81 and 0.83 for females, and 0.88 and 0.86 for males, respectively. For the maxillary second molar, the model achieved an accuracy of 88%, with female precision and recall of 0.82 and 0.91 and male precision and recall of 0.94 and 0.87, respectively. These findings support the models applicability across independent data sets and reinforce reliability for sex estimation using Random Forest classifier.

Another key advantage of AI is its ability to automate and extract meaningful patterns from complex processes. 3D geometric morphometry analysis using landmark-based shape evaluation is one such complex process which has been used to classify sex in dental and skeletal images. Softwares like “AGMT3-D” have been developed to use computer algorithms to fit landmarks automatically in 3 dimensional space which can save time and improve objectivity. (Herzlinger and Grosman 2018) This automation is particularly useful in forensic and archaeological investigations, where rapid identification is crucial.

We performed a structured literature search conducted using the Scopus database to evaluate existing research exploring the three elements, geometric morphometrics (GMM), artificial intelligence (AI), and human tooth shape for sex estimation. The search string used was: (“geometric morphometrics” OR “geometric morphometry” OR “landmark-based morphometry”) AND (“tooth shape” OR “dental morphology” OR “dentition” OR “tooth form”) AND (“artificial intelligence” OR “machine learning” OR “deep learning” OR “Random Forest” OR “support vector machine” OR “neural network”) AND (“sex estimation” OR “gender determination” OR “sex determination” OR “sexual dimorphism”) AND (“human” OR “Homo sapiens”). This search yielded 73 documents, the abstracts of which were manually screened for relevance. After excluding articles unrelated to dental morphology, studies on non-human samples, reviews, and those lacking either GMM or AI, only two studies (Oliva et al. 2021 and Bianchi et al. 2023) were found to incorporate all three essential elements: application of artificial intelligence, use of geometric morphometric landmark data, and focus on human tooth shape for sex estimation. The limited number of such studies highlights a significant gap in the literature. (Oliva et al. 2021; Bianchi et al. 2023) Oliva et al. (2021) utilized the 3D landmark data derived from premolars coupled with ANN “set aside approach” to classify sex. They showed correct classification of 90% females and 73% males with an overall accuracy of 80% in test sample. (Oliva et al. 2021) Bianchi et al. (2023) used the geometric morphometry analysis with ANN model in classifying sex based on landmark data obtained from the maxillary premolars and molars in Italian individuals (115 males and 115 females). Their method was able to classify 94% of females and 68% males accurately with an overall accuracy of 82%. (Bianchi et al. 2023).

The reason the AI models perform better than the manual methods is that AI models can handle large and complex data sets, extracting meaningful patterns from raw data without the need for predefined feature engineering. (Franco et al. 2022) RF is a decision-tree-based ensemble method, known for its robustness in handling high-dimensional data and reducing overfitting by aggregating multiple decision trees. SVM, on the other hand, is particularly effective in binary classification problems with well-defined feature spaces, as it identifies the optimal hyperplane to separate classes. RF and SVM are good at handling smaller classification data sets as they work on “dividing and conquering” principles rather than finding patterns making them excellent for classifying structured data like ours. (Borup et al. 2023) ANN and CNN, while powerful, typically require large data sets for optimal performance, and their effectiveness depends on the architecture and hyperparameter tuning, where they rely heavily on finding patterns and making connections. In cases where the data set is relatively small or lacks highly discriminative deep features, traditional machine learning models such as RF and SVM may outperform deep learning approaches.

Tree-based models (like Random Forest algorithm) are known to outperform deep learning approaches on tabular data. (Grinsztajn et al. 2022) This could be due to data set size, since it is known that machine learning algorithms, especially Random Forest, work well for tabular medium-sized data sets. As compared to deep learning-based approaches, Random Forest is not easily influenced by any noise. (Han et al. 2018) The strong performance of the Random Forest model could be attributed to its reduced risk of overfitting and its ability to learn from features, without the need for further tuning the model. (Meharie and Shaik 2020) Machine learning models, such as the RF and SVM, work better with tabulated data, whereas data and pattern extraction from images and 3D models may be more suitable for deep learning approaches like ANN. Since our data type was predominantly the coordinates extracted from the landmarks in a tabular format it explains the superiority of RF and SVM over ANN in the present analysis.

AI models are highly adaptable and can be trained on diverse data sets, making them applicable in cases, where traditional methods fall short. For instance, when skeletal remains are incomplete or degraded, AI can analyze available features, such as partial dental remains, to provide probabilistic sex classification. This adaptability extends to forensic casework, where AI can integrate multiple data sources—such as facial features, dental records, and skeletal measurements to improve classification accuracy.

The present study was conducted on a limited sample size of 120 teeth from a specific Indian subpopulation, which may not provide sufficient generalizability or robustness for artificial intelligence algorithms to achieve optimal predictive accuracy. While the pilot results indicate promising efficiency, the findings must be interpreted with caution, and further validation on a larger and more diverse population is necessary. In addition, the placement of three-dimensional geometric morphometric landmarks requires specific training and expertise, which introduces the potential for examiner-related variability. The landmarking process may thus be influenced by the individual examiner’s technique or interpretation. To overcome this limitation and enhance reproducibility, future studies should consider multi-center collaborations involving multiple investigators marking the landmarks.

## Conclusion

This study highlights the effectiveness of RF and SVM over ANN in sex estimation, suggesting that data set characteristics and feature selection play a critical role in model performance. While previous research has demonstrated the strong potential of ANN and CNN, our findings indicate that traditional machine learning models remain highly effective in specific contexts. AI offers several advantages over traditional forensic methods, such as better accuracy, automation, and objectivity. It can also handle large and complex data sets effectively. Models such as Random Forest (RF) and support vector machine (SVM) improve human identification by accurately and objectively analyzing dental shape patterns in forensic cases. Future research should explore hybrid models that combine the strengths of traditional machine learning and deep learning to further enhance sex estimation accuracy and reliability.

## Data Availability

The data sets used and/or analysed during the current study are available from the corresponding author on reasonable request.

## References

[CR1] Ajmal MA, Roberts TS, Beshtawi KR et al (2023) Sexual dimorphism in odontometric parameters using cone beam CT: a systematic review. Head Face Med 19:6 36882815 10.1186/s13005-023-00352-7 PMC9990232

[CR2] Al-Shahrani I, Dirks W, Jepson N, Khalaf K (2014) 3D-geomorphometrics tooth shape analysis in hypodontia. Front Physiol 5:1–12. 10.3389/fphys.2014.00154 24795649 10.3389/fphys.2014.00154 PMC4006061

[CR3] Anic-Milosevic S, Medancic N, Calusic-Sarac M et al (2023) Artificial neural network model for predicting sex using dental and orthodontic measurements. Korean J Orthod 53:194–204. 10.4041/kjod22.250 37226512 10.4041/kjod22.250 PMC10212777

[CR4] Bianchi I, Oliva G, Vitale G et al (2023) A semi-automatic method on a small Italian sample for estimating sex based on the shape of the crown of the maxillary posterior teeth. Healthcare 11:845. 10.3390/healthcare11060845 36981501 10.3390/healthcare11060845 PMC10048010

[CR5] Biggerstaff RH (1969) The basal area of posterior tooth crown components: the assessment of within tooth variations of premolars and molars. Am J Phys Anthropol 31:163–170. 10.1002/ajpa.1330310204 5348792 10.1002/ajpa.1330310204

[CR6] Borup D, Christensen BJ, Mühlbach NS, Nielsen MS (2023) Targeting predictors in random forest regression. Int J Forecast 39:841–868. 10.1016/j.ijforecast.2022.02.010

[CR7] Choi GPT, Chan HL, Yong R et al (2020) Tooth morphometry using quasi-conformal theory. Pattern Recognit 99:107064. 10.1016/j.patcog.2019.107064

[CR8] Esmaeilyfard R, Paknahad M, Dokohaki S (2021) Sex classification of first molar teeth in cone beam computed tomography images using data mining. Forensic Sci Int 318:110633. 10.1016/j.forsciint.2020.110633 33279763 10.1016/j.forsciint.2020.110633

[CR9] Franco A, Franco A, Porto L et al (2022) Diagnostic performance of convolutional neural networks for dental sexual dimorphism. Sci Rep 12:17279. 10.1038/s41598-022-21294-1 36241670 10.1038/s41598-022-21294-1 PMC9568558

[CR10] Grinsztajn L, Oyallon E, Varoquaux G (2022) Why do tree-based models still outperform deep learning on typical tabular data? In: Proceedings of the 36th Conference on Neural Information Processing Systems. pp 507–520

[CR11] Han T, Jiang D, Zhao Q et al (2018) Comparison of random forest, artificial neural networks and support vector machine for intelligent diagnosis of rotating machinery. Trans Inst Meas Control 40:2681–2693. 10.1177/0142331217708242

[CR12] Herzlinger G, Grosman L (2018) AGMT3-D: a software for 3-D landmarks-based geometric morphometric shape analysis of archaeological artifacts. PLoS ONE 13:1–17. 10.1371/journal.pone.0207890 10.1371/journal.pone.0207890 PMC6245792 30458049

[CR13] Khanagar SB, Vishwanathaiah S, Naik S et al (2021) Application and performance of artificial intelligence technology in forensic odontology – a systematic review. Leg Med 48:101826. 10.1016/j.legalmed.2020.101826 10.1016/j.legalmed.2020.101826 33341601

[CR14] Klingenberg CP (2011) Morphoj: an integrated software package for geometric morphometrics. Mol Ecol Resour 11:353–357. 10.1111/j.1755-0998.2010.02924.x 21429143 10.1111/j.1755-0998.2010.02924.x

[CR15] Meharie MG, Shaik N (2020) Predicting highway construction costs: comparison of the performance of random forest, neural network and support vector machine models. J Soft Comput Civil Eng 4:103–112. 10.22115/SCCE.2020.226883.1205

[CR16] Natarajan S, Ahmed J, Jose NP, Shetty S (2022) Maxillary first premolar shape (and not size) as an indicator of sexual dimorphism: a 2D geomorphometric study. F1000Res 11:433. 10.12688/f1000research.111382.3 38481535 10.12688/f1000research.111382.3 PMC10933566

[CR17] Natarajan S, Ahmed J, Shetty S et al (2024a) Geometric morphometric shape analysis of mandibular post-canine dentition. Appl Sci 14:658. 10.3390/app14020658

[CR18] Natarajan S, Junaid A, Shetty S et al (2024b) Unveiling the third dimension of tooth shape:2D versus 3D geometric morphometry of human post-canine dentition. J Oral Maxillofac Pathol 28:716–724. 10.4103/jomfp.JOMFP 39949671 10.4103/jomfp.jomfp_451_23 PMC11819640

[CR19] Nikita E, Nikitas P (2020) On the use of machine learning algorithms in forensic anthropology. Leg Med. 10.1016/j.legalmed.2020.101771 10.1016/j.legalmed.2020.101771 32795933

[CR20] Oliva G, Pinchi V, Bianchi I et al (2021) Three-dimensional dental analysis for sex estimation in the Italian population: a pilot study based on a geometric morphometric and artificial neural network approach. Healthc 10:9. 10.3390/healthcare10010009 10.3390/healthcare10010009 PMC8775125 35052173

[CR21] Porto LF, Porto LF, Lima LNC et al (2020) Estimating sex and age from a face: a forensic approach using machine learning based on photo-anthropometric indexes of the Brazilian population. Int J Legal Med 134:2239–2259. 10.1007/s00414-020-02346-5 32820357 10.1007/s00414-020-02346-5

[CR22] Robinson DL, Blackwell PG, Stillman EC, Brook AH (2002) Impact of landmark reliability on the planar Procrustes analysis of tooth shape. Arch Oral Biol 47:545–554. 10.1016/S0003-9969(02)00038-9 12208079 10.1016/s0003-9969(02)00038-9

[CR23] Rolfe S, Pieper S, Porto A et al (2021) Slicermorph: an open and extensible platform to retrieve, visualize and analyse 3D morphology. Methods Ecol Evol 12:1816–1825. 10.1111/2041-210X.13669 40401087 10.1111/2041-210x.13669 PMC12094517

[CR24] Horvath SD, Wegstein PG, Lüthi M, Blatz MB (2012) The correlation between anterior tooth form and gender - a 3D analysis in humans. Eur J Esthet Dent 22908080

[CR25] Verma R, Bhardwaj N, Singh PD et al (2021) Estimation of sex through morphometric landmark indices in facial images with strength of evidence in logistic regression analysis. Forensic Sci Int Rep 4:100226. 10.1016/j.fsir.2021.100226

[CR26] Vila-Blanco N, Vila-Blanco N, Carreira MJ et al (2020) Deep neural networks for chronological age estimation from OPG images. IEEE Trans Med Imaging 39:2374–2384. 10.1109/tmi.2020.2968765 32012002 10.1109/TMI.2020.2968765

[CR27] Wang H, Yin J, Lu P, Yu Q (2019) Three-dimensional geometric morphometric measurement and classification of maxillary central incisors. Arch Oral Biol 102:141–146. 10.1016/j.archoralbio.2019.04.005 31015059 10.1016/j.archoralbio.2019.04.005

[CR28] Yong R, Ranjitkar S, Lekkas D et al (2018) Three-dimensional (3D) geometric morphometric analysis of human premolars to assess sexual dimorphism and biological ancestry in Australian populations. Am J Phys Anthropol 166:373–385. 10.1002/ajpa.23438 29446438 10.1002/ajpa.23438

[CR29] Zhang C, Maga AM (2017) An open-source photogrammetry workflow for reconstructing 3D models Chi. AJR Am J Roentgenol 186:227–236

